# The complete chloroplast genome sequence of *Cremastra appendiculata* (Orchidaceae) revealed by next-generation sequencing and phylogenetic implication

**DOI:** 10.1080/23802359.2018.1516122

**Published:** 2018-09-22

**Authors:** Shao li Mao, Yafu Zhou, Hao Yuan, Chao Lu, Huihui Chang

**Affiliations:** a Xi’an Botanical Garden of Shaanxi Province/Institute of Botany of Shaanxi Province, Xi’an, China;; b Shaanxi Engineering Research Centre for Conservation and Utilization of Botanical Resources, Xi’an, China;; c College of Life Sciences, Shaanxi Normal University, Xi’an, China

**Keywords:** *Cremastra appendiculata*, chloroplast genome, endangered species

## Abstract

The complete chloroplast genome sequence of *Cremastra appendiculata*, a rare and endangered species of China, was determined by Illumina pair-end sequencing. The results showed that the complete plastid genome was 160,494 bp in length, containing a large single copy (LSC) of 88,249 bp and a small single copy (SSC) of 21,457 bp, which were separated by a pair of 25,394 bp inverted repeats (IRs). A total of 132 unique genes were annotated, including 86 protein coding genes, 38 tRNA genes, and 8 rRNA genes. Among these genes, 18 genes contained one or two introns. The overall GC contents of the plastid genome were 36.9%, and in the IR regions, LSC and SSC were 43.8, 34.4 and 30.9%, respectively. Maximum likelihood analysis revealed that the new sequenced species *C. appendiculata* was closer to the species in *Cattleya* and formed one clade with a high bootstrap value at the middle of the phylogenetic tree of Orchidaceae.

*Cremastra appendiculata* (D. Don) Makino, a rare and endangered plant species in China, belongs to the genus of *Cremastra* of the family Orchidaceae (Editorial Committee of Flora of China, Chinese Academy of Sciences 2009) and is contained in the Chinese pharmacopoeia (Chinese Pharmacopoeia Commission 2015). In this paper, the complete chloroplast genome sequence of *C*. *appendiculata* (Orchidaceae) was sequenced, with the aim to provide sequences data for further study and for the conservation of the rare and endangered species. The complete chloroplast genome sequence of *C. appendiculata* was submitted to GenBank under accession number MH356724.

Fresh leaves of *Cremastra appendiculata* (Orchidaceae) were collected from Mt. Qinling, Shaanxi, China (33°44′40.01″N, 107°49′15.85″E). The voucher specimens of *C*. *appendiculata* were deposited in Institute of Botany of Shaanxi Province. The complete chloroplast genome of *C*. *appendiculata* was sequenced using the Illumina HiSeq 2500 platform (Illumina, CA, USA). The complete chloroplast genome was assembled and annotation using CLC Genomics Workbench (CLC Bio, Aarhus, Denmark) and Geneious v10.1.2 (Biomatters Ltd., Auckland, New Zealand) respectively coupled with manual corrections start and stop codons, and *Elleanthus sodiroi* (GenBank: KR260986) was used as the initial reference.

The chloroplast genome sequence of *C. appendiculata* was 160,494 bp in length, which contained a pair of inverted repeat (IR) regions of 25,394 bp, a large single copy (LSC) region of 88,249 bp, and a small single copy (SSC) region of 21,457 bp. The plastid genome contains a total of 132 unique genes constituting 86 protein coding genes (PCGs), 38 transfer RNA (tRNA) genes, and 8 ribosomal RNA (rRNA) genes. Among the 132 unique genes, 12 PCGs (*rps16, atpF, rpoC1, ycf3, rps12, clpP, petB, petD, rpl16, rpl2, ndhA, ndhB*) and six tRNA (*trnG-UCC, trnK-UUU, trnV-UAC, trnL-UAA, trnI-GAU, trnA-UGC*) contained one or two introns. In addition, there are 18 genes (6 PCGs, 8 tRNA genes, and 4 rRNA genes) duplicated in the IR region. The *rps12* gene is a trans-spliced gene with a 5′ end exon in the LSC region and two duplicated 3′ end exons in the IR regions. The overall GC content was 36.9%, and in the IR regions, LSC and SSC were 43.8, 34.4 and 30.9%, respectively. The total length of all 86 PCGs was 79,245bp, and the overall GC content was 37.8%. The three most-used amino acids in C. *appendiculata* were Leu (10.4%), Ile (8.5%) and Ser (7.9%).

Phylogenetic reconstruction of Orchidaceae was performed using maximum likelihood method under the partitioned models chosen by PartitionFinder 2 (Lanfear et al. 2017), with *Iris sanguinea* and *Allium prattii* selected as outgroups. Maximum likelihood tree was constructed using RAxML 8.0 (Stamatakis 2014). The new sequenced species *C. appendiculata* was closer to *Cattleya* species and formed one clade with a high bootstrap value at the middle of the phylogenetic trees of Orchidaceae (Figure 1). The cp genome of *C*. *appendiculata* will provide new data for study and conservation of the rare and endangered species in Orchidaceae.

**Figure 1.**
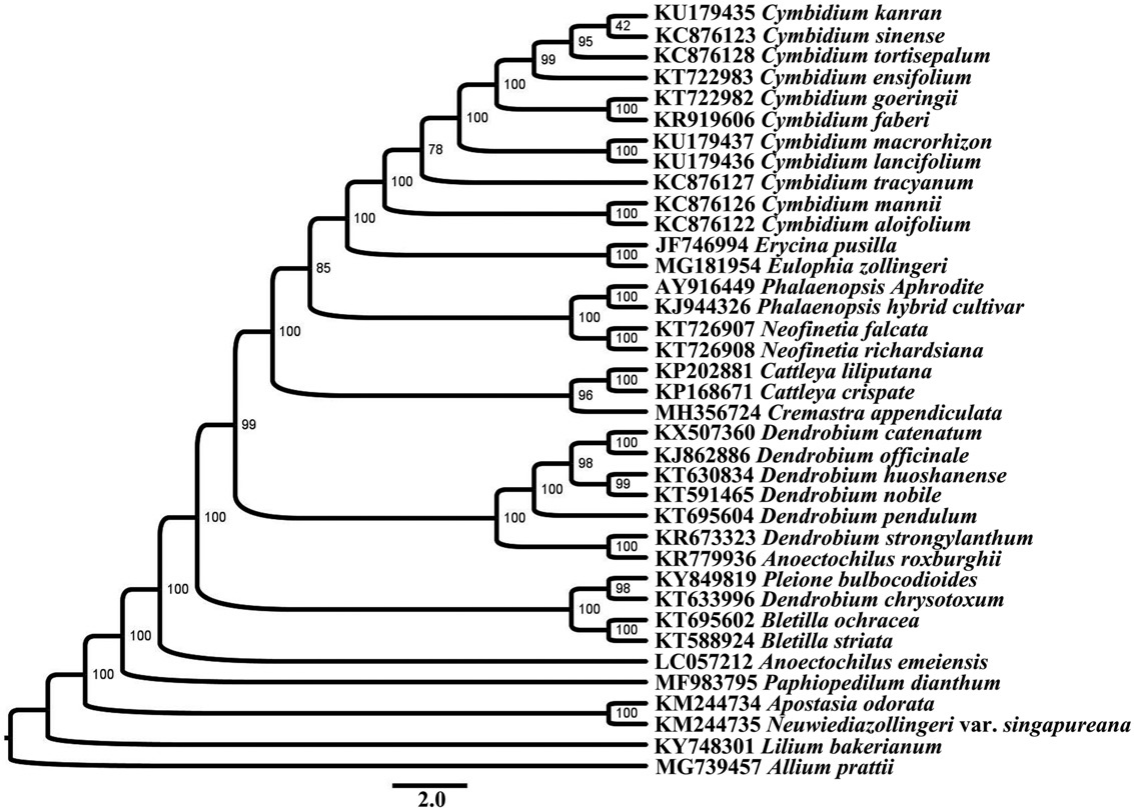
Phylogenetic reconstruction of Orchidaceae using 18 PCGs concatenated dataset.

## References

[CIT0001] Chinese Pharmacopoeia Commission 2015 Pharmacopoeia of the People's Republic of China, Vol 1. Beijing: China Medical Science Press p. 32–33.

[CIT0002] Editorial Committee of Flora of China, Chinese Academy of Sciences 2009 Flora of China, Vol 25. Beijing: Science Press p. 249–250.

[CIT0003] Lanfear R, Frandsen PB, Wright AM, Senfeld T, Calcott B 2017 PartitionFinder 2: new methods for selecting partitioned models of evolution for molecular and morphological phylogenetic analyses. Mol Biol Evol. 34:772–773. 28013191 10.1093/molbev/msw260

[CIT0004] Stamatakis A 2014 RAxML version 8: a tool for phylogenetic analysis and post-analysis of large phylogenies. Bioinformatics. 30:1312–1313. 24451623 10.1093/bioinformatics/btu033 PMC3998144

